# Effect of cortisol diurnal rhythm on emotional memory in healthy young adults

**DOI:** 10.1038/s41598-017-10002-z

**Published:** 2017-08-31

**Authors:** Mitsue Nagamine, Hiroko Noguchi, Nobuaki Takahashi, Yoshiharu Kim, Yutaka Matsuoka

**Affiliations:** 1 Institute for Liberal Arts, Tokyo Institute of Technology, 2-12-1 Ookayama, Meguro-ku, Tokyo 152-8550 Japan; 2 School of Distance Learning, Department of Human Sciences, Musashino University, 1-1-20 Shin-machi, Nishitokyo, Tokyo 202-8585 Japan; 3 Department of Clinical Psychology, Bukkyo University, 96, Kitahananobo-cho, Murasakino, Kita-ku, Kyoto 603-8301 Japan; 4 Department of Adult Mental Health, National Institute of Mental Health, National Center of Neurology and Psychiatry, 4-1-1 Ogawahigashi-cho, Kodaira, Tokyo 187-8551 Japan; 5 Division of Health Care Research, Center for Public Health Sciences, National Cancer Center Japan, 5-1-1 Tsukiji, Chuo-ku, Tokyo 104-0045 Japan

## Abstract

Few studies have investigated the relationship between cortisol diurnal rhythm and cognitive function in healthy young adults, especially for emotional memory. To address this deficiency, this study examined the effect of diurnal cortisol slope (DCS) and heart rate variability (HRV) on emotional memory. **Participants** included healthy volunteers (44 men and 23 women; mean age 20.60 yrs). Participants were shown emotionally arousing slides and were asked to return to the laboratory one week later where they were given a “surprise” memory test to examine their emotional memory retention. Participants were asked to collect saliva samples at four time points (08:00, 11:00, 15:00, and 20:00) on the experimental days; these samples were used to calculate the DCS. Moreover, HRV was measured during the experiment. The multiple linear regression analysis revealed that declarative memory ability, sleep duration, and the DCS were the final significant determinants for emotional memory enhancement (*B* = −20.41, 0.05, −48.20, *ps* < 0.05), with participants having flatter cortisol slopes showing reduced or absent emotional memory enhancement. These findings are discussed in reference to the possible effects of diurnal rhythm mechanisms of the hypothalamus-pituitary-adrenal axis and the autonomic nervous system on emotional memory.

## Introduction

Cortisol is a “stress hormone” produced by the stress-responsive hypothalamic-pituitary-adrenal (HPA) axis, the release of which affects cognitive processes such as learning and memory^1^. Previous research has examined the relationship between cortisol and learning and memory and elucidated the effects of cortisol on memory enhancement and impairment (see Wirth, 2015)^1^.

It is well known that long-term memory is enhanced by emotional events, which modulate declarative memory by activating the (nor) adrenergic system and the HPA axis^2, 3^. Over the past few decades, research has found evidence that cortisol can enhance the memory consolidation of emotional experiences, but impairs memory retrieval and working memory during emotional test situations^4^. These findings have been the result of research into the effects of acute stress; pre-learning administration of glucocorticoids^5, 6^, stress manipulation (elevated cortisol levels during encoding or consolidation)^7, 8^, and the effects of immediate post-learning stress^9^ on emotional memory. However, it is also important to understand the effects of chronic stress on emotional memory for healthcare purposes.

To understand how chronic stress affects health is very important; however, as noted by Wolf (2008)^10^, experimental human studies are not available because of ethical constraints. Therefore, most of the work in this area has relied on stimulus-based chronic stress definitions in which the target population was facing ongoing troubling circumstances^11^. From Miller’s (2007)^11^ meta-analysis and other studies^12, 13^, it was found that the dysregulation of diurnal cortisol rhythm characterized by flattened diurnal rhythm could result from exposure to chronic stressors. A typical diurnal cortisol rhythm is characterized by high levels upon waking, a steep morning rise, and subsequent steeper decline throughout the day^14^. As this pattern is considered healthy, any deviations such as a flattening of the diurnal patterns have been linked to chronic stress and poorer health outcomes^15, 16^. Therefore, a flattened cortisol diurnal rhythm is a useful indicator of a possible chronic stress diagnosis.

There have been a few findings regarding emotional regulation and cortisol diurnal rhythm from neuroimaging studies^17, 18^. These studies demonstrated the inverse relationship between diurnal cortisol rhythm and activation in the limbic brain regions (amygdala, medial prefrontal cortex, hippocampus/parahippocampus and hypothalamus), with participants who typically demonstrated greater amplitude in cortisol diurnal rhythm being found to have less brain activation in the limbic regions. In these studies, stressful and emotional stimuli were presented but memory performance was not examined. However, we speculate that memory performance, especially with emotional stimuli, would be reduced in participants with typical cortisol diurnal rhythm.

Additionally, circadian-mediated cortisol levels that is the effect of the time of day play important roles in stress reaction^19^ and cognition^20^. The varying influence of cortisol on memory due to the time of day relative to diurnal flux is connected to the relative occupancy of mineralocorticoid or glucocorticoid receptor subtypes (MR and GR subtypes, respectively), which have different affinities for glucocorticoids^21^. Although most MRs are activated throughout the day, GR activation drops from about 50% in the AM phase of the diurnal cycle to about 10% in the PM phase^21^. A review by Het *et al*. (2005)^22^ indicated that cortisol administered in the morning caused significant memory impairment, whereas in the afternoon, it resulted in a small but significant memory enhancement. Emotionality was not taken into account in these studies, although Maheu *et al*. (2005)^20^ did compare the distinct effects of a stressor applied in the AM and PM phases on long-term declarative memory for emotionally arousing and neutral material learned after the stressor. They found that stress-induced increases in salivary cortisol levels impaired the delayed free recall of emotionally arousing material in the morning group but not in the afternoon group; however, no influence of stress on memory for neutral material was found. These findings were probably caused by an overstimulation of the corticosteroid system in participants exposed to the stressor in the morning, leading to a complete saturation of both the MRs and GRs and the possible impairment of the processing capabilities of the frontal lobes, amygdala, and hippocampus^20^.

Although circadian-mediated cortisol levels play an important role in cognition^20^, to the best of our knowledge, the relationship between cortisol diurnal rhythm and memory, especially emotional memory, has not been examined in younger adults. Most studies that have investigated the relationship between cortisol diurnal rhythm and cognitive function have been conducted in aging participants and have therefore focused on cognitive aging^23–28^. These studies found that a flatter cortisol rhythm was associated with lower cognitive performance in the elderly. Abercrombie *et al*. (2004)^29^ conducted a study with middle-aged healthy and breast cancer patients, and found a link between a flatter diurnal cortisol rhythm and poorer performance in explicit memory tasks, particularly for delayed memory, only in the healthy participants. Gilpin *et al*. (2008)^30^ found that an atypical diurnal cortisol rhythm profile, which was characterized by higher cortisol levels at 22:00 compared to 14:00, were associated with cognitive deficits, particularly for novel object discrimination in participants aged between 20 and 60 years (mean age: 40 ± 12). Therefore, a flatter diurnal cortisol profile appears to be connected to cognitive impairment in middle age.

As noted above, in addition to the HPA axis, the activation of the (nor) adrenergic system has an important role in memory enhancement^2, 3^. Noradrenaline acts on amygdala beta-adrenergic receptors to modulate memory consolidation, so the activation of this region is especially important for emotional arousal^31, 32^. The findings from Segal *et al*. (2009)^33^ provided compelling evidence that noradrenergic activation underlies enhanced memory for emotional material in humans, meaning that the endogenous noradrenergic activation in response to an emotional event should predict a long-term memory of the event.

However, in our previous study, an association between the heart rate prior to an emotional event and emotional memory was found to be significant in participants with a history of post-traumatic stress disorder (PTSD)^34^. In this study, the heart rate was used as an index for an activated adrenergic system (sympathetic nervous system) during an anticipatory period of 20 seconds prior to the stimulation, which was speculated to be characterized by heightened anxiety. Interestingly, a significant association was found only for past PTSD. It is speculated that participants with a history of PTSD might have stronger associations between adrenergic activation and emotional memory compared to healthy participants. This result supported previous findings that a previous trauma was one of the risk factors influencing vulnerability to PTSD onset^35^; however, it is premature to conclude that this relationship is specific only to past PTSD subjects because of the small sample size. However, the experimental procedures that measure physiological variables such as heart rate (HR) and heart rate variability (HRV) prior to and during the emotional event are important when investigating the enhanced adrenergic nervous system in the anticipatory period.

It is also important to account for any parasympathetic system influence on emotional memory as parasympathetic activation has been found to result in rapid changes in the beat-to-beat timing of the heart compared with sympathetic activation^36^. The vagal (high-frequency [HF]) component of HRV primarily represents parasympathetic influences. Recently, there has been an increased interest in the study of HRV to clarify the dynamic adaptation-related systems in organisms^36, 37^ and because HRV is seen to be an objective measure for regulated emotional responses^38^.

The following experimental protocol developed by Cahill *et al*. (1994)^39^ has been widely used to investigate the impact of emotion on memory. Participants are shown a series of slides with an emotionally arousing accompanying narrative, and a week later, are given a surprise memory test to evaluate story retention. The emotionally arousing story consists of three phases, with the emotional elements introduced during the middle phase (Phase 2). Cahill *et al*. (1996)^40^ measured the glucose metabolic rate of the right amygdaloidal complex while subjects were viewing the emotional films and found it to be highly correlated to the number of emotional films consequently recalled. No significant correlation was found with the number of neutral films recalled. Therefore, it is plausible that an evaluation of emotional memory enhancement could be a surrogate marker for the amygdala function.

The aim of this study was to examine the effect of cortisol diurnal rhythm and heart rate variability on emotional memory in healthy young adults. To better understand the effect of cortisol diurnal rhythm on cognitive performance, the time of day at which learning took place should be taken into account. In this study, we therefore examined the time-of-day influence of cortisol diurnal rhythm on emotional memory. It was hypothesized that a flatter cortisol diurnal rhythm accentuates the memory enhancement of emotional arousal events, and this enhancement is accentuated in afternoon.

## Results

### Salivary cortisol profiles and sleep data

Salivary cortisol profiles exhibited a clear diurnal rhythm (Table 1). Analyses revealed significant main effect of diurnal rhythm (*F*(3,186) = 78.78, *p* < 0.001) and diurnal rhythm × condition interaction (*F*(3,186) = 2.81, *p* < 0.05). Simple main effect revealed that the significant cortisol decrease from 8:00 was first shown at 11:00 in both groups (*ps* < 0.001) followed by significant decrease was shown from 15:00 to 20:00 only in afternoon group (*p* < 0.001).

**Table 1:** Salivary cortisol profiles and sleep data.

	Morning (*n* = 33)		Afternoon (n = 34)		Test (*t* or χ^2^)	*p*
	*Mean*	*SD*	*Mean*	*SD*		
Salivary cortisol (nmol/l)						
8:00	15.16	10.90	15.89	8.88		
11:00	5.79	4.07	6.51	4.22		
15:00	6.25	4.02	5.67	2.90		
20:00	4.35	3.60	2.74	1.53		
Diurnal slope	−0.09	0.07	−0.13	0.05	*t*(65) = 2.79	0.01
AUC*	82.00	42.82	79.59	26.96	*t*(64) = −0.51	0.61
Wake-up time	6:50	0:45	7:10	0:30	*t*(65) = −2.11	0.04
Sleep duration	6:09	0:46	6:43	0:48	*t*(65) = −2.90	0.01

*AUC: Area under the curve. For Salivary cortisol, AUC, and Wake-up time, the statistical tests were conducted for log transformed data.

For diurnal cortisol slope, significant difference was observed in condition (*t*(65) = 2.79, *p* < 0.01). Diurnal cortisol slope was flatter in morning group than in afternoon group.

For AUC, there were no significant differences in condition or sex.

As shown in Table 1, wake-up time was earlier and sleeping duration was shorter in morning group (*t*(65) = −2.11, −2.90 *ps* < 0.05).

### Valence, Arousal, and Comprehension Levels for an Emotionally Arousing Story

The mean values for each phase are shown in Table 2. Analysis revealed significant main effects of the phase on the level of valence and arousal. A post hoc analysis showed that the phase 2 events resulted in a higher level of valence and arousal than the phase 1 and 3 events (both *ps* < 0.001). No significant main effects or interactions were observed for comprehension levels. Therefore, the participants rated the phase 2 events as being more emotional and arousing than the phase 1 events.

**Table 2:** Difference in memory score, ratings for the valence, arousal, and comprehension levels of the story, along with the HR, HRV, and SCR values by condition and sex.

		Morning				Afternoon				*df*	*F*			
		male		female		male		female			Time of Day	Sex	*df*	Phase
		*Mean*	*SD*	*Mean*	*SD*	*Mean*	*SD*	*Mean*	*SD*					
Memory score (%)	Phase1	53.08	7.97	58.44	9.62	55.68	8.51	63.99	8.12	1, 63	1.10	5.97*	2, 126	7.49**
	Phase2	60.55	7.83	60.39	7.57	63.15	7.34	65.48	9.67					
	Phase3	55.74	11.56	61.72	10.81	54.55	9.27	57.89	12.70					
Valence	Phase1	0.73	1.08	0.68	1.04	570.57	1.09	0.19	0.44	1, 63	2.00	0.69	2, 126	74.36***
	Phase2	3.08	1.96	4.18	1.50	3.17	2.01	3.58	2.08					
	Phase3	1.52	2.09	2.45	2.54	1.24	2.06	0.94	1.70					
Arousal	Phase1	0.56	0.65	0.93	1.28	0.67	1.22	0.83	0.97	1, 63	0.31	0.47	2, 126	68.90***
	Phase2	3.10	2.35	3.77	1.54	4.55	2.60	4.25	2.88					
	Phase3	1.86	1.82	3.06	2.97	2.39	2.36	2.00	2.38					
Comprehension	Phase1	9.77	0.61	9.89	0.38	9.91	0.25	10.00	0.00	1, 63	0.21	2.27	2, 126	0.94
	Phase2	9.72	0.58	9.86	0.32	9.86	0.32	10.00	0.00					
	Phase3	9.65	0.83	9.91	0.30	9.42	1.86	10.00	0.00					
HR (bpm)	Pre^*1^	70.25	8.36	73.46	8.95	72.12	9.62	72.59	17.20	1, 51	0.13	0.80	1.60, 81.8	30.24***
	Phase1	67.80	7.29	70.20	9.56	69.05	8.70	70.70	17.30					
	Phase2	66.98	7.70	68.96	8.98	67.31	8.73	69.83	17.13					
	Phase3	67.24	7.24	72.14	8.73	68.32	9.59	69.80	16.78					
LF/HF ratio	Rest^*2^	1.95	1.22	0.95	0.69	1.78	1.53	2.73	4.50	1, 50	0.52	6.30*	3, 150	6.92***
	Phase1	1.37	1.35	0.45	0.26	1.87	2.07	1.16	1.60					
	Phase2	1.67	1.48	0.58	0.44	1.80	1.68	1.17	1.09					
	Phase3	2.36	2.99	0.85	0.86	1.66	1.37	1.17	1.52					
HF-HRV (%)	Rest^*2^	34.90	16.06	52.81	21.57	39.09	16.63	40.30	21.12	1, 50	0.49	7.09*	3, 150	9.17***
	Phase1	44.85	14.84	68.15	11.60	45.76	23.91	58.08	24.13					
	Phase2	43.93	19.06	63.38	17.26	41.20	19.84	50.33	22.66					
	Phase3	42.24	22.64	57.42	19.95	44.82	21.15	55.13	21.27					
SCR (μmhos)	Pre^*1^	0.11	0.13	0.15	0.28	0.34	0.30	0.25	0.31	1, 54	0.31	0.97	3, 162	0.81
	Phase1	0.09	0.09	0.08	0.12	0.13	0.16	0.16	0.22					
	Phase2	0.07	0.09	0.06	0.11	0.06	0.07	0.18	0.23					
	Phase3	0.06	0.08	0.05	0.06	0.08	0.09	0.07	0.11					

***p < 0.001, *p < 0.05. *1 *p*re: the 20 seconds prior to phase 1, at which time a slide was shown with the following instruction: “Please watch the following slides carefully.” *2 rest: for 10 minutes prior to the pre-period. For HR, LF/HF ratio, and SCR, the statistical tests were conducted for log transformed data.

### Emotionally Influenced Memory Testing

The mean values for the memory scores are shown in Table 2. The analysis revealed significant main effects of phase (*F*(2,126) = 7.49, *p* < 0.01) and condition × phase interaction (*F*(2,126) = 3.46, *p* < 0.05). It also revealed a main effect of sex (*F*(1,63) = 5.97, *p* < 0.05). Post hoc analyses found that the memory score in phase 2 was significantly higher than that in phase 1 or phase 3 (*ps* < 0.01), indicating that emotional memory was enhanced. The simple main effect revealed a significant difference between phase 2 and phase 1 or phase 3 in the afternoon group (*p* < 0.05, *p* < 0.01) and a significant difference between phase 1 and phase 2 in the morning group (*p* < 0.05). The main effect of sex showed that enhanced emotional memory was significantly higher in males than in females but that the condition had no significant influence on emotional memory enhancement.

The mean value for the enhanced emotional memory score (±SD) was 5.49 (9.62).

### Heart Rate (HR), Heart Rate Variability (HRV), and Skin Conductance Response (SCR)

The mean values for each phase are shown in Table 2. The analysis revealed significant main effects of the phase in the HR, low-frequency [LF] component/high-frequency [HF] component (LF/HF) ratio as well as the HF component of HRV (HF-HRV). It also revealed a main effect of sex on the LF/HF ratio and HF-HRV. Post hoc analyses found that HR was significantly higher during the pre-period than in phase 1, 2, or 3 (*ps* < 0.001) and significantly higher in phase 1 than in phase 2 (*p* < 0.001). The LF/HF ratio was significantly higher in the rest period than in phase 1 or 2 (*p* < 0.001, 0.05, respectively). The HF-HRV was significantly lower during the rest period than during phase 1 (*p* < 0.001), phase 2 or 3 (*ps* < 0.05).

For sex difference, females showed significantly lower LF/HF ratios and higher HF-HRV compared to males.

### Bivariate analysis for Memory Performance and Cortisol Diurnal Slope with psychological variables or physiological variables

Pearson’s correlation coefficients are shown in Tables 3 and 4. Enhanced emotional memory was found to be significantly correlated with the Wechsler Memory Scale-Revised, the Delayed Recall Index (WMS-R) (*r* = −0.28, *p* < 0.05), the cortisol diurnal slope (*r* = −0.24, *p* = 0.05), the sleep duration (*r* = 0.26, *p* < 0.05), LF/HF ratio in phase 2 (*r* = 0.28, *p* < 0.05), and the HF-HRV in the rest period (*r* = −0.30, *p* < 0.05). The cortisol diurnal slope was found to be significantly correlated with wake-up times and sleep durations (*r* = −0.26,−0.34, *p* < 0.01, 0.05).

**Table 3:** Correlation between enhanced emotional memory and cortisol diurnal slope with psychological variables

	1		2		3		4		5		6		7		8
1. Enhanced emotional memory	—														
2. Cortisol diurnal slope	−0.24		—												
3. EPQR	−0.19		0.20		—										
4. WMS-R	−0.28	*	0.14		0.18		—								
5. STAI_S	−0.16		0.07		0.33	**	0.14		—						
6. STAI_T	−0.19		0.09		0.62	**	0.01		0.56	**	—				
7. Wake-up time	0.18		−0.26	*	0.02		−0.05		0.04		0.00		—		
8. Sleep duration	0.26	*	−0.34	**	0.06		0.03		−0.03		0.04		0.32	**	—

**p < 0.01, *p < 0.05. For EPQR, WMS-R, and Wake-up time, the statistical tests were conducted for log transformed data.

**Table 4:** Correlation between enhanced emotional memory and cortisol diurnal slope with physiological variables

	1		2		3		4		5		6		7		8		9		10		11		12		13		14		15		16		17		18		19
1. Memory*	―																																				
Cortisol																																					
2. Diurnal slope	−0.24		―																																		
3. AUC	0.05		−0.06		―																																
HR																																					
4.pre	−0.03		−0.17		0.19		―																														
5.phase1	−0.05		−0.11		0.19		0.94	***	―																												
6. phase2	−0.07		−0.09		0.23		0.93	***	0.98	***	―																										
7. phase3	−0.11		−0.02		0.24		0.91	***	0.97	***	0.99	***	―																								
HRV																																					
LF/HF ratio																																					
8. rest	0.26		−0.15		−0.06		0.28	*	0.28	*	0.29	*	0.24		―																						
9. phase1	0.22		−0.12		−0.10		0.21		0.21		0.21		0.17		0.67	***	―																				
10. phase2	0.28	*	−0.16		−0.05		0.38	**	0.38	**	0.39	**	0.33	*	0.67	***	0.68	***	―																		
11. phase3	0.15		−0.07		0.01		0.23		0.23		0.26		0.27	*	0.59	***	0.68	***	0.71	***	―																
HF(%)																																					
12. rest	−0.30	*	0.19		0.06		−0.25		−0.25		−0.26		−0.22		−0.98	***	−0.66	***	−0.67	***	−0.54	***	―														
13. phase1	−0.16		0.11		0.09		−0.20		−0.20		−0.20		−0.17		−0.67	***	−0.98	***	−0.67	***	−0.67	***	0.66	***	―												
14. phase2	−0.26		0.14		0.09		−0.39	**	−0.39	**	−0.40	**	−0.33	*	−0.65	***	−0.67	***	−0.98	***	−0.69	***	0.64	***	0.66	***	―										
15. phase3	−0.13		0.05		−0.03		−0.23		−0.23		−0.27	*	−0.28	*	−0.58	***	−0.68	***	−0.69	***	−0.99	***	0.53	***	0.67	***	0.67	***	―								
SCR																																					
16. pre	0.01		−0.07		0.24		0.14		0.13		0.14		0.16		−0.04		−0.14		−0.14		0.07		0.02		0.13		0.14		−0.10		―		―				
17. phase1	0.17		0.02		0.02		−0.02		−0.04		−0.01		0.00		0.04		−0.08		−0.06		0.15		−0.04		0.13		0.09		−0.15		0.44	**	―				
18. phase2	0.17		0.06		0.07		−0.31	*	−0.24		−0.21		−0.20		0.09		−0.09		−0.13		0.02		−0.09		0.09		0.15		−0.03		0.40	**	0.45	***	―		
19. phase3	0.15		0.06		0.05		−0.17		−0.17		−0.13		−0.12		0.17		−0.02		0.08		0.10		−0.18		0.04		−0.06		−0.09		0.35	**	0.49	***	0.66	***	―

***p < 0.001, **p < 0.01, *p < 0.05. *Memory: Enhanced emotional memory. For AUC, HR, LF/HF ratio, and SCR, the statistical tests were conducted for log transformed data.

There was no significant correlation found between enhanced emotional memory and the cortisol diurnal slope on the retrieval day (*r* = −0.05, *p* = 0.73).

### Multiple linear regression analysis for emotional memory enhancement with cortisol diurnal slope

From the results of the bivariate analysis and the repeated measures ANOVA for the physiological variables, as sex was found to be significantly associated with the LF/HF ratio and HF-HRV, only sex was entered into the multivariate model. As both sleep duration and wake-up time were significantly correlated with the cortisol diurnal slope, only sleep duration was significantly correlated with enhanced emotional memory, therefore we have entered sleep duration into the multivariate model. The cortisol diurnal slope significantly differed between the morning and afternoon groups; therefore, this condition was also entered into the multivariate model as an independent variable with the interaction term (cortisol diurnal slope × condition).

**Figure 1:**
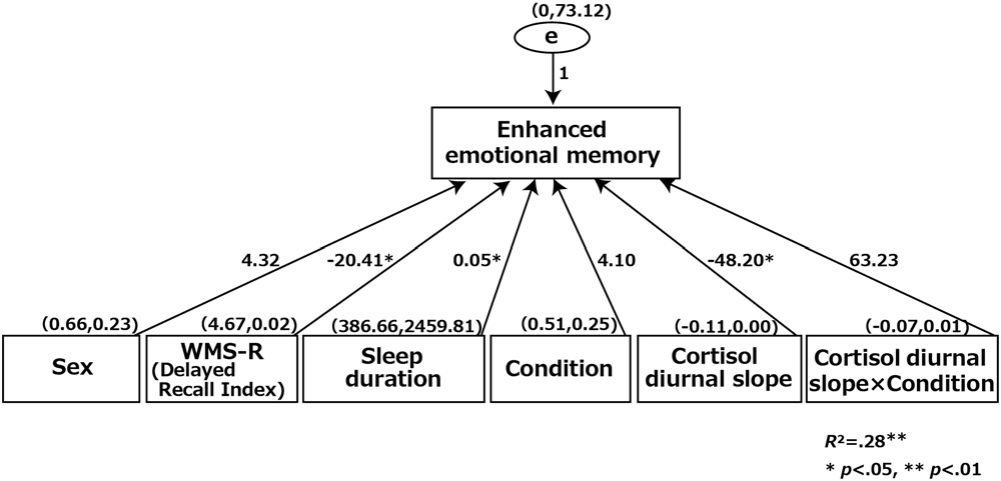
Multiple linear regression predicting enhanced emotional memory with the relevant predictors. Note: Sex and condition are entered as dichotomous variables (sex: female = 0, male = 1; condition: morning = 0, afternoon = 1). Values reported are unstandardized regression coefficients. Values in parentheses are Mean and SD.

Results of the multiple linear regression analysis are shown in Fig. 1. Values reported are unstandardized regression coefficients because it is not appropriate to standardize interaction effect in multiple regression^41^. WMS-R, sleep duration, and the cortisol diurnal slope were the final significant determinants.

## Discussion

To the best of our knowledge, the present findings are the first to show an association between cortisol diurnal rhythm and emotional memory enhancement in young healthy adults. In addition to the cortisol diurnal rhythm, the multivariate analysis revealed that the following factors were associated with emotional memory enhancement: declarative memory ability and sleep duration on the day before encoding (learning) day. First, in contrast to our hypothesis, a flatter cortisol diurnal rhythm was found to be associated with emotional memory deficits; an association that was only revealed in the cortisol diurnal rhythm on the learning day. Previous studies^27–29^ have found an association between a flatter cortisol diurnal slope and poorer memory performance, but the association with emotional memory was not investigated in these studies. Therefore, this study adds new findings to the association between a flattened cortisol diurnal slope and poorer emotional memory enhancement. A flattened diurnal cortisol slope might reflect chronic cortisol dysregulation and/or an aberrant cortisol stress response^29^. Therefore, our findings may reflect the effect of cortisol dysregulation on emotional memory deficits. However, this finding contradicts findings in previous neuroimaging studies^17, 18^ which found a healthy relationship between diurnal cortisol rhythmicity and limbic region activation. These differences in the findings might have been because of the different intensities of the experimental stimuli; that is, the emotional arousal level, as Cunningham-Bussel (2009)^18^ presented stress-related, threatening stimuli images related to the September 11^th^ World Trade Center attack, which were possibly more stressful than the stimuli used in the present study. Therefore, the relationship between cortisol diurnal rhythm and emotional memory enhancement needs to be further tested with highly intense stressful stimuli, similar to the September 11^th^ World Trade Center attack.

Several studies have investigated the memory-modulating effects of cortisol on emotionally arousing and stressful experiences, and found that there were beneficial effects to elevated cortisol levels on emotional memory at times of encoding or consolidation^3^. In this study, however, the cortisol levels did not elevate during the experiment (data not shown). In addition, in terms of subjective appraisal, that is, the level of arousal and valence in phase 2 in this study, the participants rated it lower than in previous studies (see Cahill *et al*. (1994)^39^). In our previous study with breast cancer patients with or without intrusive recollection and with a healthy control, lower rating levels compared to Cahill’s study were also found^42^. In Nagamine *et al*. (2007)^42^, it was speculated that the differences in the age of participants may be the reason for the result. However, younger participants took part in this study, and their ages were even less than the participants in Cahill’s study﻿. Therefore, these lower ratings appear not to be age-related but may reflect cultural differences. Japanese may rate the same stimuli less emotionally than people in western countries, so the subjective responses elicit a smaller increase in emotional memory enhancement; that is, the stimuli used in this study was not stressful enough for the participants.

It is speculated that a steeper cortisol diurnal rhythm showed a healthy relationship between emotional arousal and emotional memory enhancement from stimuli with a lower emotional arousal level. If the stimuli had been more stressful as in a previous study^18^, a flatter cortisol diurnal rhythm may have shown more emotional memory enhancement than a steeper cortisol diurnal rhythm. The relationship between cortisol diurnal rhythm and emotional memory enhancement might be dependent on the intensity of the stimuli. The supposed background to this relationship is the existence of an inverted-U-shaped dose-response curve between cortisol and memory^43^. However, while this inverted-U-shaped function indicates a relationship between cortisol levels and memory for emotional and neutral information, the dose-response curves may differ^44, 45^. Rimmele *et al*. (2003)^45^ suggested that for stimuli with emotional content, the dose-response curve between memory performance and cortisol levels may shift to lower values. The findings in this study may reflect the different curves between steeper and flatter cortisol diurnal rhythm groups and also those between lower and higher intensities of the experimental stimuli, that is, the emotional arousal level. Further research is needed to examine the possibility of this relationship.

It is known that psychiatric illness and post-traumatic stress disorder (PTSD) are associated with dysregulated diurnal cortisol rhythm and flatter cortisol rhythm^46^. However, it is not well known whether the flatter cortisol diurnal rhythm is a risk factor for PTSD. As mentioned in Cunningham-Bussel’s study (2009)^18^, it is important to understand the role and consequences of diurnal cortisol rhythm in both healthy adaptation and psychiatric disorders.

Second, declarative memory ability, which was assessed using the Delayed Recall Index of the Wechsler Memory Scale-Revised (WMS-R), was negatively associated with emotional memory enhancement (*r* = −0.28, *p* < 0.05). As the WMS-R test included several memory aspects but excluded emotional memory, it is plausible that emotional memory enhancement, which is defined as the difference (phase 2 − phase 1) between the memory score of the emotionally contrasted story (phase 2) and the neutral story (phase 1), was not positively correlated with WMS-R. When we separately calculated the correlation coefficients between WMS-R and the memory score in each phase, a significant correlation was only detected with phase 1, the neutral story (*r* = 0.41, *p* < 0.001), which indicated that participants who had a higher declarative memory ability had a higher score in phase1. Therefore, while emotional memory enhancement was observed in this study, the amount of enhancement was small compared to Cahill’s study(1994)^39^; however, these results were similar to those found in our previous studies^34, 42^. In our previous studies, as the correlations between WMS-R and emotional memory enhancement were not calculated, it is only speculation that the small emotional memory enhancement was due to a high phase 1 score, which indicated declarative memory ability. Further, this small emotional memory enhancement was predominant in female participants. Compared to the males, the females were found to have significantly lower LF/HF ratios, which indirectly measured sympathetic activation, and higher HF-HRV, which indirectly measured parasympathetic activation. Such sex differences in emotionally influenced memory storage have been reported in a previous study^47^ in which it was found that there was a gender-related amygdala lateralization, but there were no differences in the emotional memory scores or subjective emotional responses. The results of this study are, however, not comparable to those in Cahill’s study (2001)^47^. The reason for the small emotional memory enhancement is not clear, but one possibility is that the gender of the experimenter could have had an influence. As the experimenter in this study was female (MN), female participants may have felt more relaxed during both the rest period and the experimental procedure.

In all participants, the HF-HRV in the rest period correlated negatively and the LF/HF ratio in phase 2 correlated positively with emotional memory enhancement. That is, participants with lower parasympathetic activity showed higher emotional memory enhancement. Empirical research has found that stressful events, such as tests of cognitive ability or experimental manipulations of anxiety, worry, and fear, lead to decreased HF-HRV^48, 49^. In the present study, HF-HRV was significantly lower during the rest period than in phases 1, 2, or 3, so the anticipatory period could be seen to be the leading arousal or/and anxiety situation. The negative correlation between HF-HRV and emotional memory enhancement during the anticipatory period may reflect the effect of priming arousal on emotional memory, a result which supports the speculations of Cahill and Alkire that priming arousal could be associated with memory consolidation^9^. On the other hand, the results of the LF/HF ratio, which reflected sympathetic activation or, in other words, the activation of the (nor) adrenergic activation, indicated its important role in memory enhancement^2, 3^. As noradrenaline acts on the amygdala beta-adrenergic receptors to modulate memory consolidation, the activation of this region is especially important for emotional arousal^31, 32^. The positive correlation between the LF/HF ratio in phase 2 (emotionally arousing phase) in this study supported the previous findings of Segal *et al*. (2009)^33^ and provided compelling evidence that noradrenergic activation underlies enhanced emotional material memory in humans, indicating that the endogenous noradrenergic activation in response to an emotional event should predict a long-term memory of the event.

Third, sleep duration was found to be positively associated with enhanced emotional memory. As the recorded sleep duration in this study was from the day before the experimental day, it was not possible to clearly identify the effects of sleep duration on emotional memory consolidation. However, it has been found that sleep after learning does support memory consolidation^50^, and long-lasting memory effects of sleep deprivation have been revealed, particularly for emotional memory^51^. Wagner *et al*. (2006)^51^ and Kuriyama *et al*. (2010)^52^, who investigated the effects of sleep deprivation on emotional memory after exposure to aversive events, found that sleep deprivation facilitated the extinction of implicit fear generalization. In our study, participants who had had poor sleep on the day before the experimental day might also have had poor sleep after the experiment, which could have diminished emotional memory enhancement. In future studies, sleep parameters should also be measured on the experimental day.

In contrast to our hypothesis, no time-of-day effect was detected in this study. This was probably because the stimuli used in this study led to low level emotional arousal and no cortisol increases. To clarify the contribution of mineralocorticoid or glucocorticoid receptor subtypes (MR and GR subtypes, respectively) on emotional memory formation, cortisol secretion should be enhanced prior to the experiment using a psychosocial stress test such as the Trier Social Stress Test^53^, which induces considerable cortisol changes. Groch *et al*. (2013)^54^ found different MR and GR contributions to memory formation during sleep and theorized that insufficient MR activation, similar to GR over activation, might contribute to declarative memory impairment. Investigating the effects of MR and GR activations could provide greater insight into the functions of cortisol in emotional memory formation. Therefore, it is also important to clarify the MR and GR activations in flatter cortisol rhythms.

There are several limitations to the present study that need to be considered. First, in this study, flattened cortisol diurnal rhythm is considered to be an indicator of chronic stress; however, the examined cortisol diurnal profiles were based on sampling conducted on experimental days. Although the stimulus used in this study is not stressful enough to increase cortisol and sympathetic nervous system activation during experimentation, the cortisol diurnal profiles could have been affected by the stimulus itself. Therefore, it is premature to conclude that the flattened cortisol diurnal rhythm that represents chronic stress affects emotional memory; rather, we can only argue that a flatter cortisol diurnal rhythm was found to be associated with emotional memory deficits. Further research is needed to evaluate cortisol diurnal profiles before the experimental day as well.

Second, which is related to the first limitation, the cortisol diurnal profiles in this study were based on sampling conducted only over two days, at the learning and retrieval sessions. Therefore, multiple samples are needed to categorically establish whether the effect of diurnal cortisol rhythm dysregulation on emotional memory is different in the memory process stage.

Third, the memory task employed in this study was rated as being emotional and arousing; however, it could not be classified as a “stressful experience” because the stimuli presentation led to a gradual reduction in the SCR and an increase in HF-HRV. Therefore, a careful interpretation of our results is needed to apply the findings to trauma-related emotional memory impairment.

In summary, we report that cortisol diurnal rhythm can predict emotional memory enhancement. Participants with flatter diurnal cortisol rhythms showed a lower emotional memory enhancement for stimuli with a relatively low emotional arousal level. Thus, these findings demonstrate that cortisol is related to emotional memory consolidation both in circumstances of stress or pharmacological glucocorticoid treatment and in terms of diurnal rhythm.

## Methods

### Participants

Sixty-seven healthy, undergraduate students (44 men and 23 women) aged between 18 and 24 years (mean age: 20.6 ± 1.4) participated in this study. All participants were medication free and reported no active medical illnesses or current diagnoses for Axis I psychiatric disorders as evaluated by the Japanese version of the Mini-International Neuropsychiatric Interview^55^. This study was approved by the ethical committees of the National Center of Neurology and Psychiatry, Tokyo, Japan and the National Defense Academy, Yokosuka, Japan. The ethical committees approved the experimental procedure in accordance with the regulations of the Declaration of Helsinki, and all methods were carried out in full compliance with the approved guidelines. All participants gave written informed consent.

### Procedures

Participants reported three times to the laboratory to participate in this study. On the first day of the study, the experimental procedure was explained and written informed consent was obtained from each participant. Each of them was then interviewed and asked to complete the Mini International Neuropsychiatric Interview^55^ to ensure that they did not suffer from any current or past major psychiatric illnesses. Declarative memory ability was assessed using the Japanese version of the Wechsler Memory Scale-Revised (WMS-R)^56^. They were also asked to complete the Japanese version of the Eysenck Personality Questionnaire-Revised^57, 58^.

Health behavior such as smoking and alcohol consumption were also recorded. Participants were given cotton saliva sampling swabs (Salivette; Sarstedt, Germany) so they could assess their cortisol levels the following day. They were given written and verbal instructions for adhering to the collection protocol when the sampling swabs were supplied; they were instructed not to eat, brush their teeth, or drink liquids for at least 30 minutes before taking the sample and to refrain from intense exercise at least one hour before taking the sample. They were instructed to place the swab in their mouth and gently chew for approximately one minute to enhance the release of saliva. As accurate timing for the saliva collection was important, subjects were told that a research assistant would call about 5 minutes before the collection time to remind them to collect saliva at each designated saliva sampling time. They were also instructed to record the time they woke up as well as their sleep duration. The correct use of the sampling swabs was demonstrated and the participants practiced saliva collection in the laboratory.

Participants were randomly assigned to the morning or afternoon condition group according to their EPQ-R neuroticism score to avoid a neuroticism score difference between groups because it has been found that neuroticism is related to the cognitive processing of emotional information^59^. In total, 33 participants were assigned to the morning condition (the emotional memory experiment was conducted from 08:50 to 10:00) while 34 participants were assigned to the afternoon condition (the emotional memory experiment was conducted from 15:50 to 17:00). They were asked to have their usual sleep which meant waking up around 7:00 on the second and third experimental days to minimize any influence of sleep duration on their cortisol diurnal rhythms. About two-thirds of the participants lived in the dormitory and usually woke up at 7:00; therefore, the rest of the participants were asked to also wake up around 7:00 to reduce the differences in wake-up times among participants. The emotional memory experiment was conducted 1 to 3 weeks after the participation agreements. The participant characteristics are shown in Table 5. As shown in Table 5, there were no significant differences in psychological variables between the morning and afternoon conditions.

**Table 5:** Participant characteristics.

	Morning (*n* = 33)		Afternoon (*n* = 34)		Test (*t* or *χ* ^2^)	*p*
	*Mean*	*SD*	*Mean*	*SD*		
Age	20.64	1.52	20.56	1.37	*t*(65) = 0.22	0.83
Sex (M/F)	22/11		22/12		χ^2^(1) = 0.03	0.87
EPQR (Neuroticism)	4.67	2.77	5.32	2.86	*t*(65) = −0.88	0.38
STAI-S	36.13	6.49	37.59	7.66	*t*(64) = –0.83	0.41
STAI-T	40.18	9.85	43.50	9.37	*t*(65) = –1.41	0.16
WMS-R (Delayed Recall Index)	108.21	13.14	106.15	13.01	*t*(65) = 0.64	0.53

EPQR, Eysenck Personality Questionnaire-Revised; F, female; M, male; WMS-R, Wechsler Memory Scale-Revised; STAI-S, State-Trait Anxiety Inventory-State; STAI-T, State-Trait Anxiety Inventory-Trait. For EPQR and WMS-R, the statistical tests were conducted for log transformed data.

On the second visit, which included the emotional memory experiment (learning day), the participants arrived at the laboratory at 08:50 or 15:50. Each participant was interviewed about their sleep patterns (wake-up time, sleep duration) and asked to complete the Japanese version of Spielberger’s State-Trait Anxiety Inventory (STAI)^60, 61^. Participants were then asked to sit comfortably in a chair. Prior to viewing the slides, each participant was fitted with electrodes for continuous electrocardiography (ECG) and skin conductance response (SCR) monitoring (BIOPAC MP150 recording system; Monte System, Tokyo, Japan). They were then asked to stay quiet for the 10 minutes of the resting period (rest period).

Next, instructions were displayed 20 seconds prior to the slide presentation, and participants were told to pay attention to the slide show and the narration which was about 5 minutes long. Immediately after viewing all the slides, the participants were presented with the slides again and asked to rate their valence (on a scale of 0 [not unpleasant at all] to 10 [extremely unpleasant]), arousal (on a scale of 0 [not emotionally intense at all] to 10 [extremely emotionally intense]), and comprehension (on a scale of 0 [not understood at all] to 10 [understood completely]) for each slide. Participants were asked not to discuss the test with anyone and were instructed to return a week later.

On the third visit (retrieval day), the participants returned to the laboratory at the same time as on the second visit (at 08:50 or 15:50) and were given an unexpected memory test, consisting of five to nine multiple choice questions per slide^62^. All participants received monetary compensation (JPY 6000) for their participation.

### Memory Task

The stimulus materials (11 slides) and narrative scripts were identical to those used in a previous study^39^. Participants viewed the Japanese version of an emotionally arousing short story of about 5 minutes^63^. The story was divided into three phases: phase 1 (slides 1–4), phase 2 (slides 5–8), and phase 3 (slides 9–11). The emotionally arousing narration occurred during phase 2. Each slide was presented for 20 seconds on a 19-inch color monitor located approximately 0.7 m in front of the participant.

### Cortisol, ECG, and SCR Measurements

For the cortisol measurements, participants were asked to collect saliva samples at 4 nominated time points (08:00, 11:00, 15:00, 20:00) on the learning and retrieval days. They were asked to avoid exercising on the day of the experimental session, and were asked not to consume anything but water one hour before the start of the experiment. A small percentage of the participants (two males) were social smokers and were asked not to smoke for two weeks during the experimental sessions. Saliva samples were collected with cotton swabs (Salivette; Sarstedt, Germany) and participants were asked to store the samples in a fridge until collection. After collection, the samples were stored at −20 °C in the laboratory until analysis.

The ECG was recorded using a pair of disposable electrodes, with each electrode attached to each arm (i.e., standard limb electrocardiogram, lead I) and connected to an amplifier (ECG100C; Monte System). These signals were amplified using a bioamplifier (BIOPAC MP150; Monte System), and sampled at a rate of 1000 Hz.

The skin conductance responses (SCR) were recorded and stored using a Biopac MP150 system, and analyzed using Biopac’s software Acqknowledge 4.1. Two electrodes were attached to the pads of the index and middle fingers of the participants’ left hand using Biopac’s isotonic recording electrode gel (Gel 101). The SCR data were recorded continuously at 1000 Hz.

### Data Analysis

Saliva samples were assayed at Trier University’s Cortisol Laboratory (Trier, Germany). The cotton swabs were centrifuged at 2000 × *g* for 10 min to obtain a clear supernatant of low viscosity. Cortisol levels were determined using a competitive solid phase time-resolved fluorescence immunoassay with fluorometric end point detection. The intra-assay coefficients of variation were between 4.0% and 6.7%; the corresponding inter-assay coefficients of variation were between 7.1% and 9.0%^64^.

Based on Sephton’s study^65^, as raw value distributions were significantly skewed, the raw cortisol values were subsequently log transformed. Then a linear regression of the four cortisol values at the sampling time was calculated using SLOPE function in Microsoft Excel and the diurnal cortisol slope value was used as an index. Steeper slopes were represented by smaller beta values for the regression slope, which indicated lower cortisol levels in the evening compared with the flatter slopes (larger beta values). Additionally, the area under the curve (AUC) was calculated using the raw cortisol value for four samples with respect to ground^66^.

The basic data required for calculating of HRV is the sequence of time intervals between heart beats; this inter-beat interval time series is used to calculate the variability in the timing of the heart beat^36^. To detect the intervals, we visually inspected the raw RR interval, i.e., the time between two consecutive R-peaks detected in the QRS complexes of the continuous electrocardiographic record for the artifacts. The data were then exported as a text file to the HRV analysis software (Kubios Heart Rate Variability software, version 1.1; Biosignal Analysis and Medical Imaging Group, Department of Physics, University of Kuopio, Kuopio, Finland)^67^, and HRV analyses were conducted for the low- (LF-HRV, 0.04–0.15 Hz) and high-frequency (HF-HRV, 0.15–0.4 Hz) bands as well as for the LF/HF power ratio. The first component, LF-HRV, reflected both sympathetic and vagal activity and the HF-HRV was related to vagally mediated (parasympathetic) activity^37^. Therefore, in this study, we used the LF/HF power ratio as an indicator of sympathetic activity and the HF-HRV as an indicator of parasympathetic activity.

For each participant, the SCR was determined after viewing each slide by subtracting the baseline conductance levels (immediately before a slide was presented) from the peak changes from baseline that occurred between 1 s and 10 s after the slide was presented. An average of each participant’s responses in each phase was then calculated. The absence of a response within this window or an increase smaller than 0.02 µS was scored as a zero response^68^.

The HR and SCR were calculated in the pre-period (the 20 seconds prior to phase 1, at which time a slide was shown with the following instruction: “Please watch the following slides carefully.”) and during phases 1, 2, and 3. The HRV was calculated in the rest period (10 minutes prior to the pre-period) and during phases 1, 2, and 3.

Correct memory scores were expressed as percentages for the three different story phases as the number of questions differed in each phase^39^. Enhanced emotional memory was defined as the difference (phase 2 − phase 1) between the memory score of the emotionally contrasted story (phase 2) and the neutral story (phase 1)^69^.

Physiological and psychological data were verified for assumptions of normality and sphericity, and logarithmic transformations or Greenhouse–Geisser corrections were applied when normality or sphericity conditions were not met. Repeated analyses of variance (ANOVA) measures with condition (morning or afternoon) and sex as class variables and phase ((phase 1, 2, or 3 for psychological variables) or (pre- or rest periods, phase 1, 2, or 3 for the physiological variables)) as a repeated measure were conducted to examine the impact of emotionality on the physiological and psychological data. Simple effects and, when appropriate, Bonferroni tests were conducted on all significant physiological and psychological findings (salivary cortisol profiles, HR, LF/HF ratio, HF-HRV, valence and arousal for an emotional arousing story, memory scores) with significance accepted at *p* < 0.05. To explore the potential predictors of enhanced emotional memory, demographic (sex) and condition (morning or afternoon) variables were included in the preliminary bivariate analysis. We also calculated bivariate correlations between enhanced emotional memory and the cortisol diurnal slope with the physiological and psychological variables to explore the potential enhanced emotional memory predictors. After selecting the variables as predictors, we conducted a multiple linear regression analysis using these as the independent variables. An alpha level of 0.05 was used for all statistical tests. The data were analyzed using SPSS for Windows statistical software package (version 22.0; SPSS, Chicago, IL). The HRV data for 14 participants and SCR data for 9 participants were not obtained because of a technical error. Even after the logarithmic transformations, the AUC data for one participant was not obtained because of outliers.
